# Analytical Model for Determination of Size-Distribution of Colloidal Silver Nanoparticles from Surface Plasmon Resonance Wavelength and Dielectric Functions

**DOI:** 10.3390/nano12193474

**Published:** 2022-10-04

**Authors:** Julio Car, Nikša Krstulović

**Affiliations:** Institute of Physics, Bijenička cesta 46, 10000 Zagreb, Croatia

**Keywords:** silver nanoparticles, size of nanoparticles, surface plasmon resonance wavelength, dielectric functions, log normal size distribution, shift function

## Abstract

In this work it is shown that the size of silver nanoparticles in a colloidal solution can be determined only from the wavelength of the surface plasmon resonance and material and medium dielectric functions. The size dependence of dielectric functions of silver nanoparticles becomes noticeable in nanoparticles which are smaller than 30 nm in size, which is in accordance with Mie scattering theory applicability. The novelty of this work is in the development of an analytical model for the determination of the size of silver nanoparticles derived from applying shift functions to the UV-Vis spectra, resulting in well-known characteristic diameters of log-normal size distribution function. The purpose of these shift functions is the reconstruction of experimental UV–Vis spectra from simulated ones based on the Beer–Lambert law and log-normal distribution function in order to find the mode diameters of colloidal silver nanoparticles. The introduction of Lagrangian analogue of extinction cross section explains the redshift constant characteristic for given nanoparticle material and the size distribution of nanoparticles. Therefore, the size determination of colloidal silver nanoparticles is possible only through UV–Vis spectroscopy.

## 1. Introduction

Nanoplasmonics is a highly researched field due to the wide range of applications which it enables: sensorics [1], energetics [2], nanomedicine [3], bio-imaging [4], photothermal effects [5], and optoelectronics [6]. It has been shown that the size of nanoparticles plays a significant role in determining various properties of nanoparticles: optical [7], mechanical [8], thermal [9], electrical [10], magnetic [11], transport [12], reactive [13], and catalytic [14]. These properties are directly related to the efficiency of nanoparticles in respective applications. Although a number of research papers have dealt with the problematics of size determination of colloidal nanoparticles, they often referred to the use of more than one piece of experimental data. The majority of papers in this matter refer to the profound work of Haiss et al. [15], where the problem of the size and concentration of gold nanoparticles is solved by several methods. Through size correction of Drude dielectric functions and Mie scattering theory, Haiss et al. showed that the SPR wavelength vs the diameter of colloidal gold nanoparticles has exponential form. Additionally, in the same work it was shown that the ratio of SPR absorbance to absorbance at 450 nm vs logarithm of nanoparticles’ diameter follows linear law. However, both methods rely on fitted parameters which are not physically determined and are limited to monodispersing spherical colloidal nanoparticles. Mansour et al. [16] demonstrated that gold nanoparticles’ size distribution can be determined from extinction spectroscopy by solving the inverse problem using Mie theory and a non-negative least square algorithm. The sensitivity of width and position of the surface plasmon resonance (SPR) was clearly demonstrated by the size and standard deviation of size distribution of normally distributed colloidal gold nanoparticles. However, matrix calculations are necessary and are time-consuming. Pashkov et al. [17] used a machine learning algorithm for the inverse design of structural parameters of colloidal solutions of nanoparticles from their optical characteristics. By varying the structural parameters to achieve the smallest norm of predicted to experimental UV–Vis spectra, one can predict the size and shape of nanoparticles from their optical spectra. Since the theoretical training set which the machine learning algorithm uses for the reconstruction of experimental UV–Vis spectra is prone to systematic errors, this approach is still in its early stage. Martinez et al. [18] implemented a methodology based on principal components’ analysis and showed that gold nanoparticles’ size can be determined from the SPR peak position of UV–Vis spectra. This approach is purely mathematical since it is based on linear combinations of variables orthogonal to each other constructed in such a way as to minimize the variance of data set. It is also based on fitting parameters which do not have a physical background or whose physical background is rather unknown. In this work an analytical model for the size determination of colloidal silver nanoparticles based on only one experimental parameter is presented. The developed model proposes that only the position of the SPR determines the mode diameter of colloidal silver nanoparticles because dependence on width of size distribution can also be expressed as a function of the SPR wavelength. Motivation for this work was further simplification of the method for determining the nanoparticles’ size [19,20] without the use of electron microscopy, atomic force microscopy, or X-ray diffraction techniques [21]. An additional motivation is to develop such procedures for silver instead of gold nanoparticles on which a number of works already exist. The theoretical framework for this work includes Mie scattering theory, log-normal size distribution and shift functions for the reconstruction of experimental UV–Vis spectra. The novelty of this work is in the avoidance of an excessive number of input experimental parameters and through derivations reduction to only one by assuming that the material and medium dielectric functions are already known. The fundamental question of the interrelation between the SPR wavelength and the size of nanoparticles is solved through the Lagrangian analogue of extinction cross section. It explains the origin of the redshift constant emerging in shift functions of UV–Vis spectra. Furthermore, the width of the plasmon band in UV–Vis spectra due to the width of size distribution of colloidal silver nanoparticles is accounted for in the standard deviation of log-normal size distribution and shown to be a function of the SPR wavelength and redshift constant. This model is not limited by the monodispersity of colloidal nanoparticles, but allows a wide distribution of nanoparticle sizes. Although the spherical morphology of nanoparticles is assumed by default, the question of different morphologies is still under consideration. Calculations by a developed analytical model are provided for 79 independent samples of colloidal silver nanoparticles with relevant data obtained by UV–Vis spectroscopy. SPR wavelengths and diameters are reported for each sample by the Paramelle et al. [22] and NanoComposix [23,24,25]. 

## 2. Computational Methods

The reconstruction of UV–Vis spectra using the Beer–Lambert law and log-normal size distribution function results in spectra with an SPR wavelength shifted from an experimental SPR wavelength. The narrow plasmon bandwith appears due to the neglect of higher than dipole terms of extinction cross section and redshift effect due to the depolarization of the electromagnetic wave with the increase in size of nanoparticles as well as the size dependence of dielectric functions. Therefore, a simple equation [20] to simulate UV–Vis spectra given by (1) fails to reconstruct the experimental UV–Vis spectra:

(1) $$A\left(\lambda \right)\ln\left(10\right)=\sum_{j}\sigma_{j}\left(D_{j},\lambda ,\varepsilon \left(\lambda \right)\right)\cdot c_{j}\left(D_{j}\right)\cdot l$$

**Figure 1:**
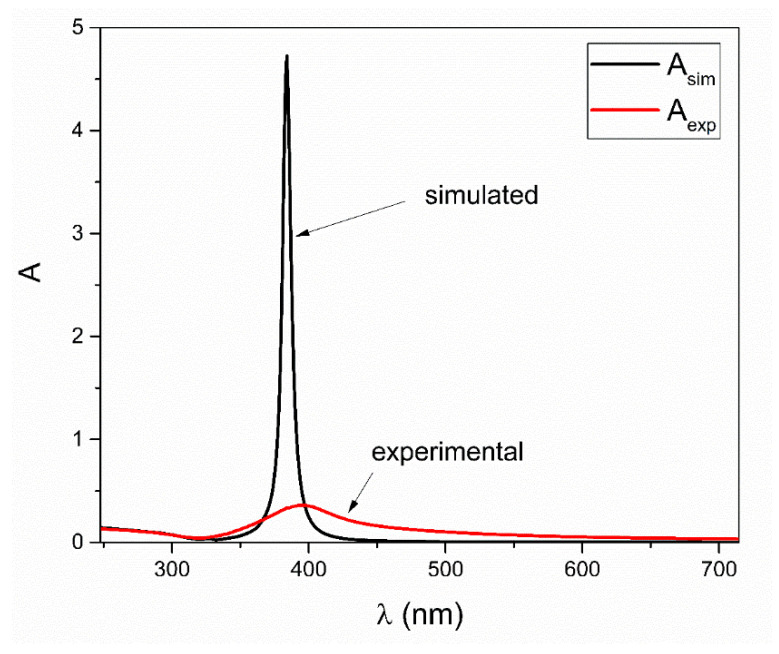
Comparison between experimental and simulated UV–Vis spectra obtained by using Equation (1). Adapted with permission from Ref. [19], Springer Nature, 2021.

Nevertheless, integral areas under both experimental and simulated UV–Vis spectra are practically the same, as can be seen in Figure 1. In order to explain why simulated UV–Vis spectra based on a summation of products of extinction cross sections, concentrations and the optical path length in a given size range of nanoparticles differ from experimental UV–Vis spectra, shift functions are introduced. These shift functions are hypothesized to have the following form:

(2) $$f_{n}\left(D_{j}\right)=K_{n}\frac{D_{j}{}^{n}}{D_{j}{}^{2}+{\left(\Delta \lambda \right)}^{2}}$$

where n is a natural number and j is the index of nanoparticles’ sizes in range $D_{j}=\left[D_{\min}..D_{\max}\right]$. 

**Figure 2:**
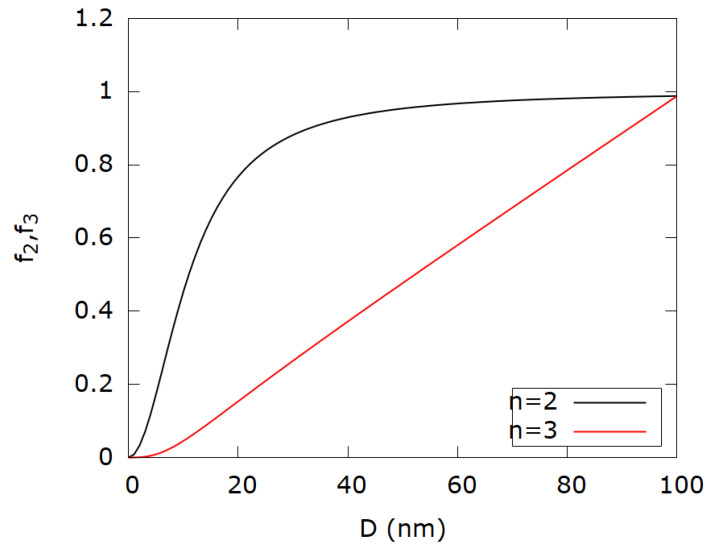
Shift functions f_2_ and f_3_ for constants K_2_ = 1 and K_3_ = 0.01, respectively.

Functions of form (2) are hypothesized to be generating functions for the redshift of scattered wavelengths due to radiative damping terms. Emphasis is put on the shift function for n = 2 because it accounts for the near SPR dissipation of plasmon energy for three reasons. The first reason is that plasmon oscillations can be explained by a damped harmonic oscillator for which the second-order differential equation is well known. By analogy, the mass-spring system is represented by electron density (mass) oscillation under the influence of incident electromagnetic waves (external force) by restoring the Coulomb force (spring) due to positive ions. The oscillatory solution for the position of electrons in time has a decaying amplitude due to damping terms caused by the increasing size of nanoparticles (radiative loss). From spectral analysis, it is well known that oscillation with decaying amplitude in frequency space corresponds to the intensity profile which has Lorentzian shape [26]. It is obtained by means of Fourier analysis. The second reason is the fact that for weakly dissipating nanoparticles, in a Rayleigh limit scattering cross section with the use of Drude permittivity the model is exactly Lorentzian [27]. The third reason is the fact that it was shown that radiative decay rate for plasmonically active metal nanoparticles increases quadratically with the size of nanoparticles [28], which was accounted for in the Lorentzian used for shift function in this work. The shift function for n = 2, $f_{2}\left(D_{j}\right)=K_{2}\frac{D_{j}{}^{2}}{D_{j}{}^{2}+{\left(\Delta \lambda \right)}^{2}}$ has Lorentzian shape in variable λ but for fixed Δλ it accounts for the size dependence of the redshift. The reason for the fixed Δλ is the shift of the experimental SPR from the theoretical one. Shift function $f_{3}\left(D\right)$ shows linear behavior. Figure 2 shows shift functions depending on the order n. The shift function $f_{1}\left(D\right)$ was not taken into consideration since it does not produce any relevant results. The purpose of shift functions is to account for the redshift of UV–Vis spectra due to the size and size distribution of colloidal nanoparticles. Since shift functions multiply on the right hand side of the Beer–Lambert law given by (1), their physical interpretation is as follows: the product of concentration and shift function results in the effective concentration of nanoparticles. Since this analysis is focused only on the SPR peak, the following can be written:

(3) $$A\left(\lambda_{\mathrm{SPR}}\right)\ln\left(10\right)=\sum_{j}\sigma_{j}\left(D_{j},\lambda_{\mathrm{SPR}},\varepsilon \left(\lambda_{\mathrm{SPR}}\right)\right)\cdot c_{\mathrm{eff}}\left(D_{j}\right)\cdot l$$

where

(4) $$c_{j}\left(D_{j}\right)=\frac{N_{j}\left(D_{j}\right)}{V_{\mathrm{liq}}}=\frac{V_{\mathrm{eff}}}{V_{j}V_{\mathrm{liq}}}\cdot p\left(D_{j}\right)=\frac{6V_{\mathrm{eff}}}{V_{\mathrm{liq}}{\pi D}_{j}^{3}}\cdot p\left(D_{j}\right)$$

(5) $$p\left(D_{j}\right)=\frac{1}{2}\left[\mathrm{erf}\left(\frac{\ln\left(D_{j}+\Delta D\right)-\ln\left(D_{x}\right)}{\sqrt{2}s}\right)-\mathrm{erf}\left(\frac{\ln\left(D_{j}\right)-\ln\left(D_{x}\right)}{\sqrt{2}s}\right)\right]$$

(6) $$c_{\mathrm{eff}}\left(D_{j}\right)=c_{j}\left(D_{j}\right)\cdot f_{n}\left(D_{j}\right)$$

The research objective is to establish the relation between characteristic diameters corresponding to log-normal size distribution function (volume average diameter, mode diameter, model diameter, and median diameter) via shift functions given by (2). This can be done by observing how shift functions for particular n affect the effective number concentration. It has been shown that by demanding the conservation of total concentration, the following equations can be written:

(7) $$K_{2}\frac{1}{D_{m}}\cdot \frac{1}{D_{m}^{2}+{\left(\Delta \lambda \right)}^{2}}=\frac{1}{{\langle D\rangle }^{3}}$$

(8) $$\frac{K_{3}}{{\left(D_{m}e^{\frac{s^{2}}{2}}\right)}^{2}+{\left(\Delta \lambda \right)}^{2}}=\frac{1}{{\langle D\rangle }^{3}}$$

Combining (7) and (8), one can write:

(9) $$K_{3}=K_{2}\frac{1}{D_{m}}\cdot \frac{{\left(D_{m}e^{\frac{s^{2}}{2}}\right)}^{2}+{\left(\Delta \lambda \right)}^{2}}{D_{m}^{2}+{\left(\Delta \lambda \right)}^{2}}$$

As Mansour et al. [1] qualitatively showed in their work, standard deviation in size distribution of colloidal nanoparticles has aneffect on both the width and position of the SPR peak. In formalism developed in this work, it can be shown that the following relation holds:

(10) $$e^{\frac{s^{2}}{2}}=\sqrt[{3}]{K_{2}}\cdot {\left(\frac{\lambda_{0}}{\lambda_{\mathrm{SPR}}}\right)}^{3}$$

It shows that standard deviation ($e^{\frac{s^{2}}{2}}$) consists of both the redshift ($K_{2}$) and width of the plasmon band ($\frac{\lambda_{0}}{\lambda_{\mathrm{SPR}}}$).

Thorough analysis starting from

(11) $$D_{M}^{3}=K_{2}\frac{D_{m}^{5}}{D_{m}^{2}+{\left(\Delta \lambda \right)}^{2}}$$

finally leads to the analytical expression for size of colloidal silver nanoparticles:

(12) $$D_{m}=\sqrt[{3}]{K_{2}}\frac{\lambda_{0}}{\lambda_{\mathrm{SPR}}}\frac{\Delta \lambda }{\sqrt{{\left(\frac{\lambda_{\mathrm{SPR}}}{\lambda_{0}}\right)}^{9}-1}}$$

The derivation procedure which leads to result (12) is shown in Supplementary Material. As can be seen from formula (12), the mode diameter of colloidal silver nanoparticles depends solely on the SPR wavelength, theoretical resonance wavelength ($\lambda_{0}=384$ nm for silver nanoparticles in water) originating from the Frohlich condition for the SPR ($\varepsilon_{1}=-2\varepsilon_{m}$), and their difference. Constant $K_{2}$ is a function of the SPR wavelength and material and medium dielectric functions which remains to be shown. The remaining open question is how to evaluate the redshift constant $K_{2}$ from the shift function $f_{2}$ in terms of the SPR wavelength and dielectric functions of material and medium. Furthermore, physical interpretation of the given constant is necessary as it is crucial in the determination of the mode (most frequent) diameter of nanoparticles in the colloidal solution. In order to explain the origin and meaning of constant $K_{2}$, we examined two functions, $\sigma_{e}^{+}\left(D\right)$ and $\sigma_{e}^{-}\left(D\right)$. These functions have the following form, respectively:

(13) $$\sigma_{e}^{+}\left(D\right)=\sigma_{a}\left(D\right)+\sigma_{s}\left(D\right)=3\pi^{2}\varepsilon_{m}^{3/2}\frac{\varepsilon_{2}}{{\left(\varepsilon_{1}+2\varepsilon_{m}\right)}^{2}+\varepsilon_{2}^{2}}\frac{D^{3}}{\lambda }+\frac{2}{3}\pi^{5}\varepsilon_{m}^{2}\frac{{\left(\varepsilon_{1}-\varepsilon_{m}\right)}^{2}+\varepsilon_{2}^{2}}{{\left(\varepsilon_{1}+2\varepsilon_{m}\right)}^{2}+\varepsilon_{2}^{2}}\frac{D^{6}}{\lambda^{4}}$$

(14) $$\sigma_{e}^{-}\left(D\right)=\sigma_{a}\left(D\right)-\sigma_{s}\left(D\right)=3\pi^{2}\varepsilon_{m}^{\frac{3}{2}}\frac{\varepsilon_{2}}{{\left(\varepsilon_{1}+2\varepsilon_{m}\right)}^{2}+\varepsilon_{2}^{2}}\frac{D^{3}}{\lambda }-\frac{2}{3}\pi^{5}\varepsilon_{m}^{2}\frac{{\left(\varepsilon_{1}-\varepsilon_{m}\right)}^{2}+\varepsilon_{2}^{2}}{{\left(\varepsilon_{1}+2\varepsilon_{m}\right)}^{2}+\varepsilon_{2}^{2}}\frac{D^{6}}{\lambda^{4}}$$

The first function $\sigma_{e}^{+}\left(D\right)$ is well known from the Mie scattering theory as an extinction cross section for an electromagnetic wave on a spherical obstacle in the dipole limit and it is the sum of absorption and scattering cross sections which were derived using [29]. The second function $\sigma_{e}^{-}\left(D\right)$ is the authors’ construction of a function which is the difference between absorption and scattering cross sections. The reason for this definition of $\sigma_{e}^{-}\left(D\right)$ will become clear and justified in the next sections. For a shorter notation, one can use $K_{1}^{*}=3\pi^{2}\varepsilon_{m}^{3/2}\frac{\varepsilon_{2}}{{\left(\varepsilon_{1}+2\varepsilon_{m}\right)}^{2}+\varepsilon_{2}^{2}}$ and $K_{2}^{*}=\frac{2}{3}\pi^{5}\varepsilon_{m}^{2}\frac{{\left(\varepsilon_{1}-\varepsilon_{m}\right)}^{2}+\varepsilon_{2}^{2}}{{\left(\varepsilon_{1}+2\varepsilon_{m}\right)}^{2}+\varepsilon_{2}^{2}}$ so these equations become:

(15) $$\sigma_{e}^{+}\left(D\right)=K_{1}^{*}\frac{D^{3}}{\lambda }+K_{2}^{*}\frac{D^{6}}{\lambda^{4}}$$

(16) $$\sigma_{e}^{-}\left(D\right)=K_{1}^{*}\frac{D^{3}}{\lambda }-K_{2}^{*}\frac{D^{6}}{\lambda^{4}}$$

$\sigma_{e}^{+}\left(D\right)$ and $\sigma_{e}^{-}\left(D\right)$ are given as a function of variable D because the wavelength of examination is fixed and equals the SPR. Analysis of both functions will be made comparatively for a real colloidal solution of silver nanoparticles with an SPR wavelength 395 nm and mode diameter 19.3 nm [19]. Dielectric functions for the given SPR wavelength are taken from [30].

Furthermore, since the extinction efficiency which is given as a ratio of extinction and geometric cross sections $\frac{\sigma_{e}\left(D\right)}{\sigma_{g}\left(D\right)}$ is physically relevant for describing the optical properties of colloidal solutions of nanoparticles, comparative analysis of $\frac{\sigma_{e}^{+}\left(D\right)}{\sigma_{g}\left(D\right)}$ and $\frac{\sigma_{e}^{-}\left(D\right)}{\sigma_{g}\left(D\right)}$ will be made too. Here, the geometric cross section is defined as $\sigma_{g}\left(D\right)=\frac{\pi }{4}D^{2}$.

### 2.1. Case of $\sigma_{e}^{+}\left(D\right)$ and $\sigma_{e}^{-}\left(D\right)$

Function $\sigma_{e}^{+}\left(D\right)$ which represents the sum of absorption and scattering cross sections is a rising function in variable D in the range D>0 and therefore is not expected to have a global maximum. However, the plot of $\sigma_{e}^{+}\left(D\right)$ for the fixed SPR wavelength and corresponding dielectric functions which are dependent on the same SPR wavelength, shows interesting behavior. The calculation of first and second derivatives confirms the existence of a global minimum. If $\frac{\partial \sigma_{e}^{+}\left(D\right)}{\partial D}=0$ is calculated, the following results are obtained:

(17) $$D_{\min}=-\sqrt[{3}]{\frac{1}{2}}\sqrt[{3}]{\frac{K_{1}^{*}}{K_{2}^{*}}}\lambda_{\mathrm{SPR}}$$

(18) $$\sigma_{e}^{+}\left(D_{\min}\right)=-\frac{1}{4}\frac{K_{1}^{*}{}^{2}}{K_{2}^{*}}\lambda_{\mathrm{SPR}}^{2}$$

The physical interpretation of the obtained results is challenging. The very fact that function $\sigma_{e}^{+}\left(D\right),$ which represents the extinction cross section according to Mie scattering theory, has a negative global minimum for a negative diameter is surprising. Although negative values imply no physical meaning of obtained results, it will be shown that (17) has an important role in deriving constant $K_{2}$ needed for the main formula (12). The question is how in reality for the given size of nanoparticles, does the UV–Vis spectrum form and is it connected to the shape of function $\sigma_{e}^{+}\left(D\right)$? Mathematically speaking, the very existence of the extreme of function $\sigma_{e}^{+}\left(D\right)$ implies the condition on the SPR wavelength from which the size of colloidal nanoparticles can be determined.

On the other hand, the constructed function $\sigma_{e}^{-}\left(D\right)$ which represents the difference between the absorption and scattering cross sections is both a rising and falling function in the range D>0. The plot of $\sigma_{e}^{-}\left(D\right)$ shows the existence of a global maximum which differential calculus confirms. If $\frac{\partial \sigma_{e}^{-}\left(D\right)}{\partial D}=0$ is calculated, the following results are obtained:

(19) $$D_{\max}=\sqrt[{3}]{\frac{1}{2}}\sqrt[{3}]{\frac{K_{1}^{*}}{K_{2}^{*}}}\lambda_{\mathrm{SPR}}$$

(20) $$\sigma_{e}^{-}\left(D_{\max}\right)=\frac{1}{4}\frac{K_{1}^{*}{}^{2}}{K_{2}^{*}}\lambda_{\mathrm{SPR}}^{2}$$

**Figure 3:**
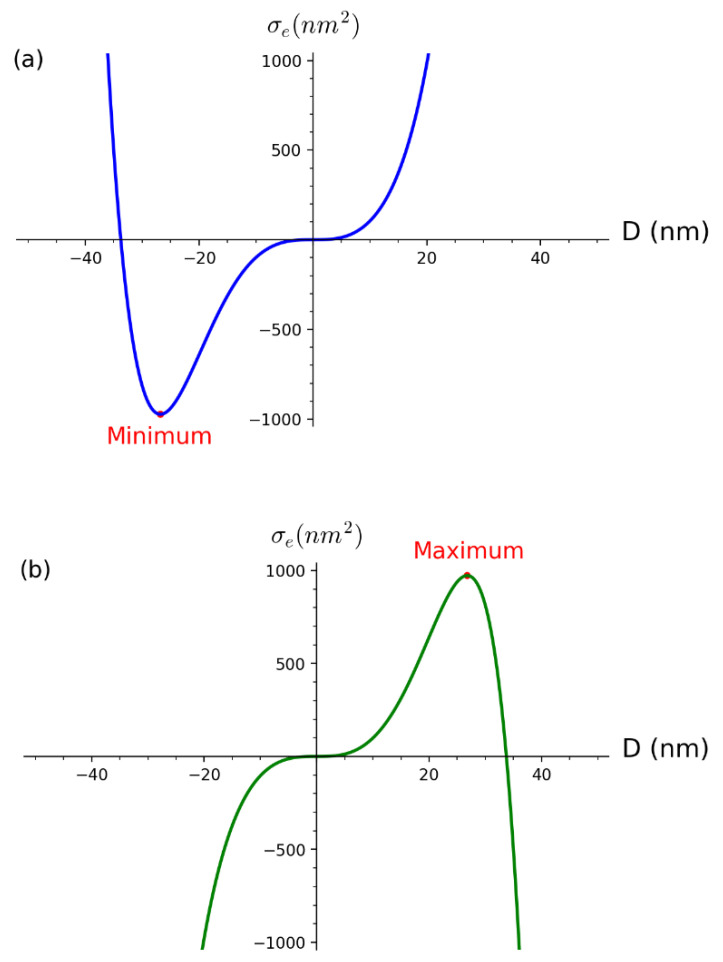
Functions (**a**) $\sigma_{e}^{+}\left(D\right)$ and (**b**) $\sigma_{e}^{-}\left(D\right)$ as functions of diameter D. On the plots are indicated global minimum for $\sigma_{e}^{+}\left(D\right)$ and global maximum for $\sigma_{e}^{-}\left(D\right)$.

These results are positive and symmetrical to ones obtained for $\sigma_{e}^{+}\left(D\right)$. Although the definition of $\sigma_{e}^{-}\left(D\right)$ as a difference between absorption and scattering cross sections is artificially constructed, it does have a physical meaning relevant to the relation between the size of colloidal nanoparticles and the SPR wavelength. The question is how to justify that diameter $D_{\max}$ is related to the SPR wavelength and why it occurs for the extreme value of $\sigma_{e}^{-}\left(D\right)$? Physical proof is as follows: since the function $\sigma_{e}^{-}\left(D\right)$ has a maximum, it means that for $D_{\max}$ the difference between the absorption and scattering cross sections is maximum. This is only possible at the SPR (meaning that $D_{\max}$ must be related to the SPR wavelength). In other words, the size of synthesized colloidal nanoparticles represents $D_{\max}$ for which the SPR wavelength is unequivocally defined by (19). Figure 3 shows both $\sigma_{e}^{+}\left(D\right)$ and $\sigma_{e}^{-}\left(D\right)$ with the indicated global minimum and maximum, respectively. Both functions are obtained for fixed $K_{1}^{*}=40$, $K_{2}^{*}=64151$ which corresponds to the SPR wavelength $\lambda_{\mathrm{SPR}}=395$ nm.

The construction of $\sigma_{e}^{-}\left(D\right)$ resembles to the definition of Lagrangian in classical mechanics as a difference between kinetic and potential energy of a physical system rather than a sum, so by analogy, we look for the interpretation of $\sigma_{e}^{-}\left(D\right)$ in terms of $\sigma_{e}^{+}\left(D\right)$. The symmetry of the extreme points of $\sigma_{e}^{-}\left(D\right)$ and $\sigma_{e}^{+}\left(D\right)$ gives weight to, at first sight, an irrelevant negative diameter and global minimum of $\sigma_{e}^{+}\left(D\right)$ since they really represent the point at which the extinction efficiency is maximized, which is the property of surface plasmon resonance.

### 2.2. Case of $\frac{\sigma_{e}^{+}\left(D\right)}{\sigma_{g}\left(D\right)}$ and $\frac{\sigma_{e}^{-}\left(D\right)}{\sigma_{g}\left(D\right)}$

Functions $\frac{\sigma_{e}^{+}\left(D\right)}{\sigma_{g}\left(D\right)}$ and $\frac{\sigma_{e}^{-}\left(D\right)}{\sigma_{g}\left(D\right)}$ have the following forms:

(21) $$\frac{\sigma_{e}^{+}\left(D\right)}{\sigma_{g}\left(D\right)}=\frac{4}{\pi }\frac{D}{\lambda }\left(K_{1}^{*}+K_{2}^{*}{\left(\frac{D}{\lambda }\right)}^{3}\right)$$

(22) $$\frac{\sigma_{e}^{-}\left(D\right)}{\sigma_{g}\left(D\right)}=\frac{4}{\pi }\frac{D}{\lambda }\left(K_{1}^{*}-K_{2}^{*}{\left(\frac{D}{\lambda }\right)}^{3}\right)$$

By examining ratios of $\frac{\sigma_{e}^{+,-}\left(D\right)}{\sigma_{g}\left(D\right)}$, similar results are obtained as for $\sigma_{e}^{+,-}\left(D\right)$. Qualitatively, $\frac{\sigma_{e}^{+}\left(D\right)}{\sigma_{g}\left(D\right)}$ has global minimum while $\frac{\sigma_{e}^{-}\left(D\right)}{\sigma_{g}\left(D\right)}$ has global maximum. Quantitatively, differential calculus gives the same results for diameters as before in terms of parameters but different factors. Specifically, for $\frac{\sigma_{e}^{+}\left(D\right)}{\sigma_{g}\left(D\right)}$ we obtain:

(23) $$D_{\min}=-\sqrt[{3}]{\frac{1}{4}}\sqrt[{3}]{\frac{K_{1}^{*}}{K_{2}^{*}}}\lambda_{\mathrm{SPR}}$$

(24) $$\frac{\sigma_{e}^{+}\left(D_{\min}\right)}{\sigma_{g}\left(D_{\min}\right)}=-\frac{3}{\pi }\sqrt[{3}]{\frac{1}{4}}\frac{K_{1}^{*}\sqrt[{3}]{K_{1}^{*}}}{\sqrt[{3}]{K_{2}^{*}}}$$

and similarly for $\frac{\sigma_{e}^{-}\left(D\right)}{\sigma_{g}\left(D\right)}$ we obtain:

(25) $$D_{\max}=\sqrt[{3}]{\frac{1}{4}}\sqrt[{3}]{\frac{K_{1}^{*}}{K_{2}^{*}}}\lambda_{\mathrm{SPR}}$$

(26) $$\frac{\sigma_{e}^{-}\left(D_{\max}\right)}{\sigma_{g}\left(D_{\max}\right)}=\frac{3}{\pi }\sqrt[{3}]{\frac{1}{4}}\frac{K_{1}^{*}\sqrt[{3}]{K_{1}^{*}}}{\sqrt[{3}]{K_{2}^{*}}}$$

**Figure 4:**
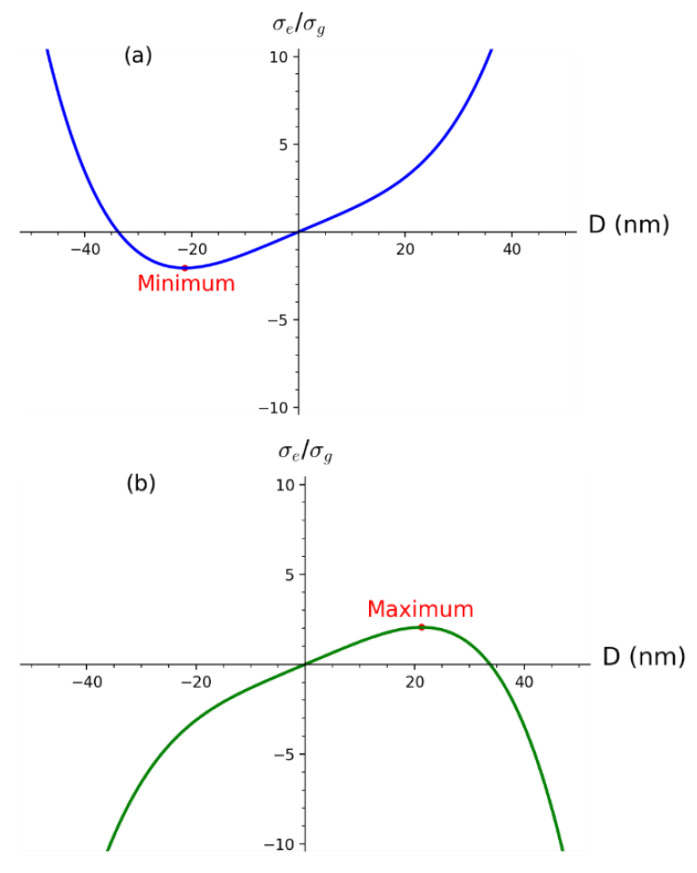
Functions (**a**) $\frac{\sigma_{e}^{+}\left(D\right)}{\sigma_{g}\left(D\right)}$ and (**b**) $\frac{\sigma_{e}^{-}\left(D\right)}{\sigma_{g}\left(D\right)}$ as functions of diameter D. Global minimum for $\frac{\sigma_{e}^{+}\left(D\right)}{\sigma_{g}\left(D\right)}$ and global maximum for $\frac{\sigma_{e}^{-}\left(D\right)}{\sigma_{g}\left(D\right)}$ is indicated.

The symmetry of obtained results for $\frac{\sigma_{e}^{+}\left(D\right)}{\sigma_{g}\left(D\right)}$ and $\frac{\sigma_{e}^{-}\left(D\right)}{\sigma_{g}\left(D\right)}$ implies the same physical interpretation as for the case of $\sigma_{e}^{+}\left(D\right)$ and $\sigma_{e}^{-}\left(D\right)$. It must be emphasized that the points of minimum and maximum for efficiency do not depend on the SPR wavelength explicitly although the diameter does. It means that the efficiency has its maximum depending on constants $K_{1}^{*}$ and $K_{2}^{*}$ which are functions of dielectric constants (which in turn are functions of the wavelength). Physically, it will be shown that the shift in extinction efficiency $\frac{\sigma_{e}^{+}\left(D\right)}{\sigma_{g}\left(D\right)}$ by shift function $f_{2}\left(D_{\max}\right)$ reconstructs experimental UV–Vis spectra while $\frac{\sigma_{e}^{-}\left(D\right)}{\sigma_{g}\left(D\right)}$ gives a physical explanation for the of symmetrical $D_{\max}$ instead of $D_{\min}$. Figure 4 shows both functions $\frac{\sigma_{e}^{+}\left(D\right)}{\sigma_{g}\left(D\right)}$ and $\frac{\sigma_{e}^{-}\left(D\right)}{\sigma_{g}\left(D\right)}$ with the indicated global minimum and maximum, respectively. Both functions are obtained for fixed $K_{1}^{*}=40$, $K_{2}^{*}=64151$ which correspond to the SPR wavelength $\lambda_{\mathrm{SPR}}=395$ nm. Notice the less steep functions in Figure 4 than functions in Figure 3.

### 2.3. Derivation of Constant $K_{2}$

Prior to derivation of analytical expression for $K_{2}$, its physical interpretation is necessary. The constant $K_{2}$ is obtained from the shift function $f_{2}\left(D\right)$ and represents the redshift constant of simulated UV–Vis spectra into experimental UV–Vis spectra due to the size distribution of colloidal nanoparticles. The most profound way to observe the effect of shift function $f_{2}\left(D\right)$ on simulated UV–Vis spectra is by focusing on its SPR peak. The product of the theoretical SPR peak with shift function $f_{2}\left(D\right)$ reconstructs the experimental SPR peak. The following equation can be written for the redshift of the theoretical SPR peak into the experimental SPR peak:

(27) $$\frac{\sigma_{e}^{+}\left(D_{\max},\lambda_{0}\right)}{\sigma_{g}\left(D_{\max}.\lambda_{0}\right)}=\frac{\sigma_{e}^{+}\left(D_{\max},\lambda_{\mathrm{SPR}}\right)}{\sigma_{g}\left(D_{\max}.\lambda_{\mathrm{SPR}}\right)}\cdot K_{2}\frac{D_{\max}{}^{2}}{D_{\max}{}^{2}+{\left(\Delta \lambda \right)}^{2}}$$

Notice that in the shift function $D_{\max}$ is squared, meaning that even $D_{\min}$ although negative can be used, which resolves the physical question of its origin. Since in theory $\lambda_{0}\to \lambda_{\mathrm{SPR}}$, it implies the following identity:

(28) $$K_{2}\frac{D_{\max}{}^{2}}{D_{\max}{}^{2}+{\left(\Delta \lambda \right)}^{2}}=1$$

from which $K_{2}$ can be derived and with use of (25) it equals:

(29) $$K_{2}=1+{\left(\frac{\Delta \lambda }{D_{\max}}\right)}^{2}=1+4^{\frac{2}{3}}{\left(\frac{K_{2}^{*}}{K_{1}^{*}}\right)}^{\frac{2}{3}}{\left(\frac{\Delta \lambda }{\lambda_{\mathrm{SPR}}}\right)}^{2}$$

Since shift functions are dimensionless, it means that the redshift constant $K_{2}$ is also dimensionless or alternatively has dimensions $\left[\frac{\mathrm{nm}}{\mathrm{nm}}\right]$ which can be interpreted as the shift in wavelength Δλ by increment of diameter ΔD.

The analytical model for the sizes of colloidal silver nanoparticles given by (12) has the following final form:

(30) $$D_{m}=\sqrt[{3}]{1+4^{\frac{2}{3}}{\left(\frac{K_{2}^{*}}{K_{1}^{*}}\right)}^{\frac{2}{3}}{\left(\frac{\Delta \lambda }{\lambda_{\mathrm{SPR}}}\right)}^{2}}\frac{\lambda_{0}}{\lambda_{\mathrm{SPR}}}\frac{\Delta \lambda }{\sqrt{{\left(\frac{\lambda_{\mathrm{SPR}}}{\lambda_{0}}\right)}^{9}-1}}$$

where $K_{1}^{*}=3\pi^{2}\varepsilon_{m}^{3/2}\frac{\varepsilon_{2}}{{\left(\varepsilon_{1}+2\varepsilon_{m}\right)}^{2}+\varepsilon_{2}^{2}}$ and $K_{2}^{*}=\frac{2}{3}\pi^{5}\varepsilon_{m}^{2}\frac{{\left(\varepsilon_{1}-\varepsilon_{m}\right)}^{2}+\varepsilon_{2}^{2}}{{\left(\varepsilon_{1}+2\varepsilon_{m}\right)}^{2}+\varepsilon_{2}^{2}}$. All characteristic diameters of the log-normal size distribution (volume average, number average, model, median) as well as the size distribution itself can be determined with the use of (12) and (30).

## 3. Results and Discussion

In this section tables with reported SPR wavelengths and diameters from Paramelle et al. [22] for colloidal silver nanoparticles are reported. The additional column has the diameter values obtained by the analytical model (30) developed in this work (modeled diameter). 

**Figure 5:**
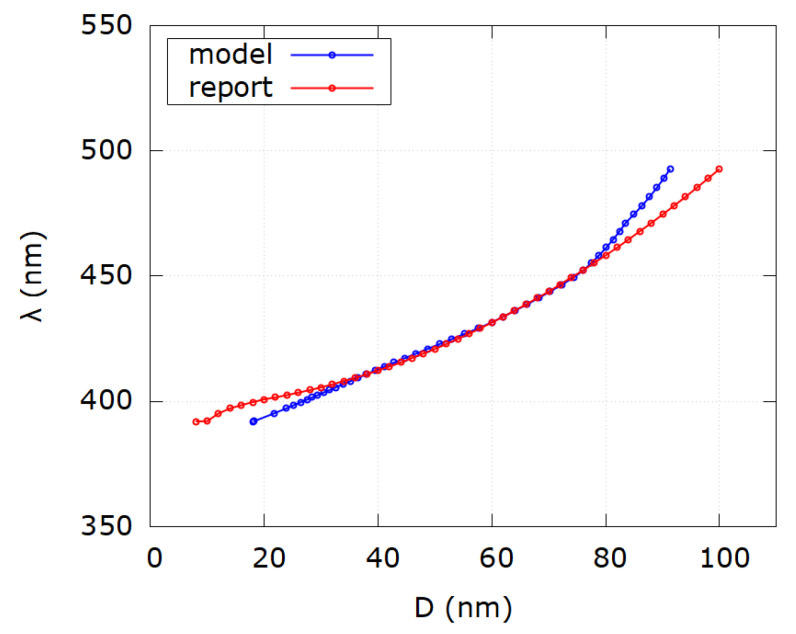
Comparison of SPR wavelength as a function of diameter for modeled values (blue) and reported values (red) from Table 1. Notice discrepancy for very small and very big diameter values.

Table 1 shows that the relative error between reported and modeled diameter values is significant for samples 1–11, which correspond to the sizes of nanoparticles smaller than 30 nm. This result is not unexpected and is in accordance with the validity of the Mie scattering theory as written in [31]. Furthermore, since for all calculations bulk silver dielectric functions were used, the reason for the discrepancy for D<30 nm can be the size dependence of dielectric functions due to the free mean path of electrons in silver. Discrepancy also exists for samples 42–47, which corresponds to the sizes of nanoparticles larger than 80 nm. Although not significant as for sizes D<30 nm, the reason for it might be neglect of higher multipole terms of Mie scattering theory since all calculations were done in the dipole limit. Absolute average relative error for all samples is $\left|\Delta \right|=\left|\frac{D-D_{m}}{D_{m}}\right|\cdot 100\%=9.7\%$. However, if only the range (30–80) [30..80] nm is considered, the absolute average relative error becomes |Δ|=1.3%. Figure 5 shows the comparison of SPR wavelengths as a function of diameter for reported and modeled values according to Table 1. Functional behavior shows good matching in the range (30–80) nm while outside that range discrepancy occurs.

**Figure 6:**
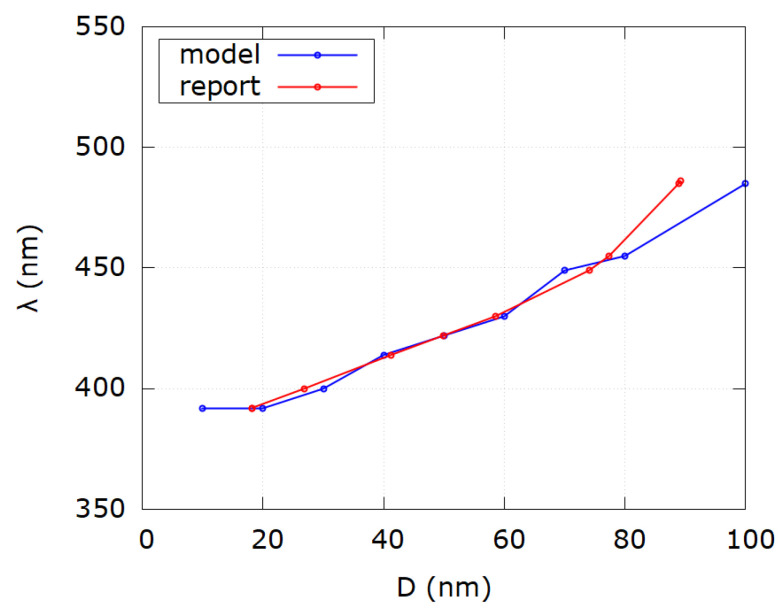
Comparison of SPR wavelength as a function of diameter for modeled values (blue) and reported values (red) from first 10 samples of Table 2. Notice discrepancy for very small and very big diameter values.

**Figure 7:**
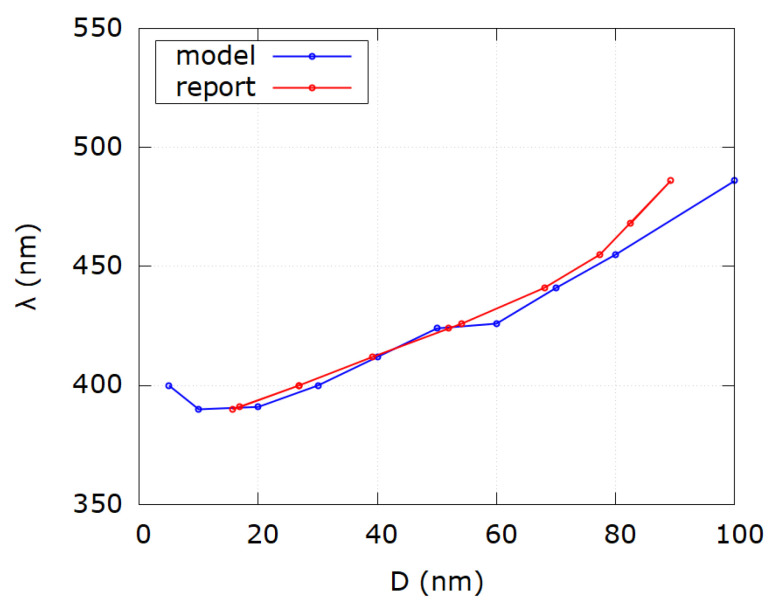
Comparison of SPR wavelength as a function of diameter for modeled values (blue) and reported values (red) from samples 11–21 of Table 2. Notice discrepancy for very small and very big diameter values.

In Table 2 the discrepancy between reported and modeled diameters is again seen in the range up to 30 nm for the same physical reasons as mentioned before. For reported diameters of 100 and 200 nm the difference is obvious, which can be explained by the same formalism of multipole expansion of extinction cross sections in the Mie theory. Absolute average relative error is mainly influenced by those values which show the biggest discrepancy. Absolute average relative error for all samples is $\left|\Delta \right|=\left|\frac{D-D_{m}}{D_{m}}\right|\cdot 100\%=25.9\%$. However, by excluding samples outside the range (30–80) nm, absolute average relative error becomes |Δ|=4.62%. Figure 6 shows the comparison of SPR wavelengths as a function of diameter for reported and modeled values according to the first 10 samples in Table 2. Discrepancy between reported and modeled values exists for diameters less than 20 nm and larger than 80 nm. Figure 7 shows the comparison of SPR wavelengths as a function of diameter for reported and modeled values according to samples 11–21 of Table 2. As for Figure 6, discrepancy between reported and modeled values occurs for diameters less than 20 nm and larger than 80 nm.

**Figure 8:**
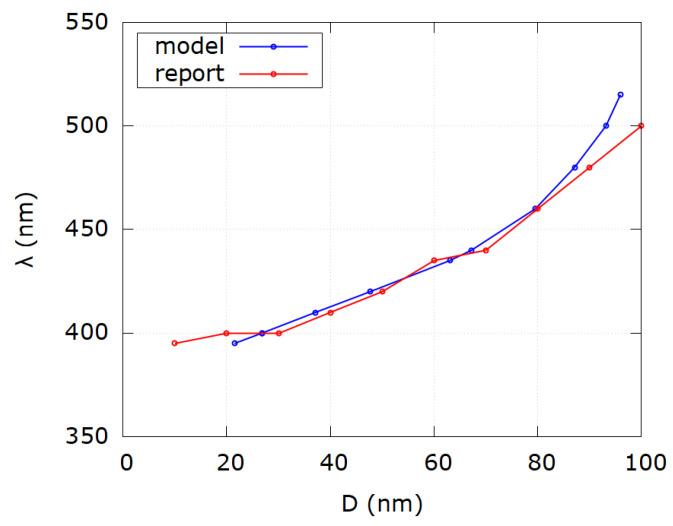
Comparison of SPR wavelength as a function of diameter for modeled values (blue) and reported values (red) from Table 3. Notice discrepancy for very small and very big diameter values.

In Table 3 the same procedure was done as for previous data and the same conclusions can be drawn. Again for reported diameters 10 and 20 nm as well as 100 and 110 nm, the discrepancy is obvious. Absolute average relative error for all samples is $\left|\Delta \right|=\left|\frac{D-D_{m}}{D_{m}}\right|\cdot 100\%=18.6\%$. However, for the range (30–90) nm it becomes |Δ|=5%. It must be, nevertheless, mentioned that values of SPR wavelengths are reported as whole numbers unlike in Table 1, which affects modeled diameter values. Reported diameters are also reported as whole values which in practice is not the case. Furthermore, the analytical model for sizes of colloidal silver nanoparticles developed in this work outputs mode diameters of colloidal nanoparticles while reported diameters are given as average values obtained from TEM images, which might be an additional reason for the observed discrepancy. Figure 8 shows the comparison of SPR wavelengths as a function of diameter for reported and modeled values according to Table 3. It can be seen that as a general rule, discrepancy appears for sizes less than 20 nm and larger than 90 nm for reasons elaborated earlier.

## 4. Conclusions

In this work, an analytical model for the determination of mode diameters of colloidal silver nanoparticles from UV–Vis spectroscopy was developed. It relies only on the SPR wavelength and dielectric functions of the nanoparticle material and medium. Derivation of the model involved the Mie theory in the dipole limit, log-normal size distribution and shift functions for explanation of the discrepancy between simulated and experimental UV–Vis spectra of nanoparticles with a given size range. The use of shift functions $f_{2}$ and $f_{3}$ and the characteristic diameters of log-normal size distribution lead to the final expression of the mode diameter of colloidal silver nanoparticles. Since analytical expression depends on the redshift constant $K_{2}$, a discussion was had in order to physically interpret its origin. The use of two symmetric functions $\sigma_{e}^{+}\left(D\right)$ and $\sigma_{e}^{-}\left(D\right)$ where the first represents the extinction cross section and the second the difference between absorption and scattering cross sections resulted in a global minimum and a global maximum, respectively, with symmetrical extreme points. Since extinction efficiency given as a ratio of extinction and geometric cross sections is more relevant for the description of UV–Vis spectra, $\frac{\sigma_{e}^{+}\left(D\right)}{\sigma_{g}\left(D\right)}$ and $\frac{\sigma_{e}^{-}\left(D\right)}{\sigma_{g}\left(D\right)}$ were examined. The Lagrangian-like form of $\frac{\sigma_{e}^{-}\left(D\right)}{\sigma_{g}\left(D\right)}$ was discussed and the importance of global maximum emphasized. The main conclusion is that the diameter value $D_{\max}$ which corresponds to this maximum represents the diameter for which the difference between the absorption and the scattering cross section is maximized; thisoccurs at the surface plasmon resonance. Finally, the derivation of redshift constant $K_{2}$ was made showing that it is a function of the experimental SPR wavelength, theoretical SPR wavelength and dielectric functions. The main discrepancy between reported and modeled diameters comes from the size dependence of dielectric function for sizes less than 30 nm as well as the neglect of higher multipole terms of the extinction cross section for sizes larger than 80 nm.

## Figures and Tables

**Table 1:** Reported SPR wavelengths λ_SPR_, reported diameters D, modeled diameters D_m_ and references of 47 samples of colloidal silver nanoparticles.

Sample i	SPR Wavelength λ_SPR_	Reported Diameter D	Modeled Diameter D_m_	Reference
1	392	8	18.1	[22]
2	392.1	10	18.3	[22]
3	395.2	12	21.7	[22]
4	397.2	14	23.9	[22]
5	398.5	16	25.2	[22]
6	399.7	18	26.5	[22]
7	400.8	20	27.6	[22]
8	401.6	22	28.5	[22]
9	402.5	24	29.4	[22]
10	403.5	26	30.4	[22]
11	404.5	28	31.5	[22]
12	405.6	30	32.6	[22]
13	406.8	32	33.9	[22]
14	408.1	34	35.2	[22]
15	409.4	36	36.5	[22]
16	410.8	38	37.9	[22]
17	412.3	40	39.5	[22]
18	413.9	42	41.1	[22]
19	415.5	44	42.8	[22]
20	417.3	46	44.8	[22]
21	419.1	48	46.7	[22]
22	420.9	50	48.7	[22]
23	422.9	52	50.8	[22]
24	424.9	54	53.0	[22]
25	427	56	55.3	[22]
26	429.2	58	57.7	[22]
27	431.5	60	60.0	[22]
28	433.8	62	62.0	[22]
29	436.2	64	64.1	[22]
30	438.7	66	66.2	[22]
31	441.3	68	68.3	[22]
32	443.8	70	70.2	[22]
33	446.7	72	72.4	[22]
34	449.5	74	74.4	[22]
35	452.3	76	76.1	[22]
36	455.3	78	77.5	[22]
37	458.3	80	78.8	[22]
38	461.4	82	80.1	[22]
39	464.6	84	81.3	[22]
40	467.9	86	82.4	[22]
41	471.2	88	83.5	[22]
42	474.6	90	84.9	[22]
43	478.1	92	86.4	[22]
44	481.6	94	87.7	[22]
45	485.3	96	89.0	[22]
46	489	98	90.2	[22]
47	492.8	100	91.3	[22]

**Table 2:** Reported SPR wavelengths λ_SPR_, reported diameters D, modeled diameters D_m_ and references of 21 samples of colloidal silver nanoparticles.

Sample i	SPR Wavelength λ_SPR_	Reported Diameter D	Modeled Diameter D_m_	Reference
1	392	10	18.1	[23]
2	392	20	18.1	[23]
3	400	30	26.8	[23]
4	414	40	41.2	[23]
5	422	50	49.8	[23]
6	430	60	58.5	[23]
7	449	70	74.0	[23]
8	455	80	77.4	[23]
9	485	100	88.9	[23]
10	486	200	89.2	[23]
11	400	5	26.8	[24]
12	390	10	15.7	[24]
13	391	20	16.9	[24]
14	400	30	26.8	[24]
15	412	40	39.2	[24]
16	424	50	52.0	[24]
17	426	60	54.2	[24]
18	441	70	68.0	[24]
19	455	80	77.4	[24]
20	486	100	89.2	[24]
21	468	200	82.5	[24]

**Table 3:** Reported SPR wavelengths λ_SPR_, reported diameters D, modeled diameters D_m_ and references of 11 samples of colloidal silver nanoparticles.

Sample i	SPR Wavelength λ_SPR_	Reported Diameter D	Modeled Diameter D_m_	Reference
1	395	10	21.5	[25]
2	400	20	26.8	[25]
3	400	30	26.8	[25]
4	410	40	37.1	[25]
5	420	50	47.7	[25]
6	435	60	63.0	[25]
7	440	70	67.2	[25]
8	460	80	79.5	[25]
9	480	90	87.1	[25]
10	500	100	93.2	[25]
11	515	110	95.9	[25]

## Data Availability

All data and derivations are available on request.

## Supplementary Materials

The following supporting information can be downloaded at: https://www.mdpi.com/article/10.3390/nano12193474/s1.

## Abbreviations

SPR	surface plasmon resonance
λ	wavelength
$\lambda_{0}$	theoretical SPR wavelength
$\lambda_{\mathrm{SPR}}$	experimental SPR wavelength
Δλ	difference between experimental and theoretical SPR wavelength
A(λ)	absorbance
D	general diameter of nanoparticles
$D_{j}$	j-th diameter in range $\left[D_{\min}..D_{\max}\right]$
ΔD	increment of diameter $D_{j+1}-D_{j}$
ε(λ)	general dielectric functions
$\sigma_{j}\left(D_{j},\lambda ,\varepsilon \left(\lambda \right)\right)$	extinction cross sections of nanoparticles with diameter $D_{j}$
$\varepsilon_{1}$	real part of material dielectric function
$\varepsilon_{2}$	imaginary part of material dielectric function
$\varepsilon_{m}$	real part of medium dielectric function
$c_{j}\left(D_{j}\right)$	concentration of nanoparticles with diameter $D_{j}$
l	optical path length
n	order of shift function (natural number)
$K_{n}$	redshift constant of order n
$f_{n}\left(D_{j}\right)$	shift function of order n
$c_{\mathrm{eff}}\left(D_{j}\right)$	effective concentration of nanoparticles with diameter $D_{j}$
$N_{j}\left(D_{j}\right)$	number of nanoparticles with diameter $D_{j}$
$p\left(D_{j}\right)$	log-normal probability function
$V_{j}\left(D_{j}\right)$	volume of nanoparticle with diameter $D_{j}$
$V_{\mathrm{eff}}$	effective crater volume
$V_{\mathrm{liq}}$	volume of liquid
s	standard deviation of log-normal size distribution
$D_{m}$	mode diameter of log-normal size distribution
$D_{M}$	model diameter of log-normal size distribution
$D_{x}$	median diameter of log-normal size distribution
〈D〉	volume average diameter of log-normal size distribution
$\sigma_{a}\left(D\right)$	absorption cross section
$\sigma_{s}\left(D\right)$	scattering cross section
$\sigma_{e}^{+}\left(D\right)$	extinction cross section
$\sigma_{e}^{-}\left(D\right)$	difference between $\sigma_{a}\left(D\right)$ and $\sigma_{s}\left(D\right)$
$\sigma_{g}\left(D\right)$	geometric cross section
$K_{1}^{*}$	absorption term of extinction cross section
$K_{2}^{*}$	scattering term of extinction cross section
$\frac{\sigma_{e}^{+}\left(D\right)}{\sigma_{g}\left(D\right)}$	extinction efficiency
$\frac{\sigma_{e}^{-}\left(D\right)}{\sigma_{g}\left(D\right)}$	efficiency of difference between $\sigma_{a}\left(D\right)$ and $\sigma_{s}\left(D\right)$
$D_{\min}$	minimal diameter value of function $\sigma_{e}^{+}\left(D\right)$ or $\frac{\sigma_{e}^{+}\left(D\right)}{\sigma_{g}\left(D\right)}$
$\sigma_{e}^{+}\left(D_{\min}\right)$	global minimum of function $\sigma_{e}^{+}\left(D\right)$
$D_{\max}$	maximal diameter value of function $\sigma_{e}^{-}\left(D\right)$ or $\frac{\sigma_{e}^{-}\left(D\right)}{\sigma_{g}\left(D\right)}$
$\sigma_{e}^{-}\left(D_{\max}\right)$	global maximum of function $\sigma_{e}^{-}\left(D\right)$
$\frac{\sigma_{e}^{+}\left(D_{\min}\right)}{\sigma_{g}\left(D_{\min}\right)}$	global minimum of function $\frac{\sigma_{e}^{+}\left(D\right)}{\sigma_{g}\left(D\right)}$
$\frac{\sigma_{e}^{-}\left(D_{\max}\right)}{\sigma_{g}\left(D_{\max}\right)}$	global maximum of function $\frac{\sigma_{e}^{-}\left(D\right)}{\sigma_{g}\left(D\right)}$
Δ	average relative error
