# Supplementary material for: Analytical Model for Determination of Size-Distribution of Colloidal Silver Nanoparticles from Surface Plasmon Resonance Wavelength and Dielectric Functions
Source: Nanomaterials (Basel). 2022 Oct 4;12(19):3474. doi: 10.3390/nano12193474 (PMC9565655; doi:10.3390/nano12193474)
Supplement: Supplementary file 1 [file nanomaterials-12-03474-s001.zip › nanomaterials-1916949-supplementary.pdf]

nanomaterials

MDPI

*Supplementary Material*

# Analytical Model for Determination of Size-Distribution of Colloidal Silver Nanoparticles from Surface Plasmon Resonance Wavelength and Dielectric Functions

**Julio Car and Nikša Krstulović ***

Institute of Physics, Bijenička cesta 46, 10000 Zagreb, Croatia
* Correspondence: niksak@ifs.hr

Through use of shift functions $f_2(D)$ and $f_3(D)$ on concentration $c_j(D)$ given by (6) and conservation of total concentration, characteristic diameters of log-normal size distribution are obtained. Namely, those are mode (most frequent) diameter $D_m$, volume average diameter $\langle D\rangle = D_m e^{-\frac{s^2}{2}}$ and model diameter $D_M = D_m e^{\frac{s^2}{2}}$. Concretly, from conservation of total concentration volume average diameter $\langle D\rangle$ is obtained, by multiplying concentration with shift function $f_2(D)$ mode diameter $D_m$ is obtained and by multiplying concentration with shift function $f_3(D)$ model diameter $D_M$ is obtained. From that, we start with following system of equations:

$$K_2 \frac{D_m^2}{D_m^2 + (\Delta\lambda)^2} = e^{\frac{3}{2}s^2} \tag{S1}$$

$$K_3 \frac{1}{(D_M)^2 + (\Delta\lambda)^2} = \frac{1}{(\langle D\rangle)^3} \tag{S2}$$

Combining the two equations (S1) and (S2), relation between redshift constants is obtained:

$$K_3 = K_2 \frac{1}{D_m} \frac{\left(D_m e^{\frac{s^2}{2}}\right)^2 + (\Delta\lambda)^2}{D_m^2 + (\Delta\lambda)^2} \tag{S3}$$

In order to calculate shift of concentration by function $f_3(D)$, (S3) is used and following must be satisfied:

$$K_3 \frac{\left(D_m e^{\frac{s^2}{2}}\right)^3}{\left(D_m e^{\frac{s^2}{2}}\right)^2 + (\Delta\lambda)^2} = K_2 \frac{D_m^2 e^{\frac{3}{2}s^2}}{D_m^2 + (\Delta\lambda)^2} \tag{S4}$$

Expression (S4) must be by definition equal to:

$$K_2 \frac{D_m^2 e^{\frac{3}{2}s^2}}{D_m^2 + (\Delta\lambda)^2} = \left(\frac{D_M}{\langle D\rangle}\right)^3 \tag{S5}$$

Before continuing derivation, one can show that (S1) can be rewritten as:

$$e^{\frac{1}{2}s^2} = K_2 \cdot \left(\frac{\lambda_0}{\lambda_{SPR}}\right)^3 \tag{S6}$$

Rewriting equation (S5), one obtains:

$$D_M^3 = K_2 \frac{D_m^5}{D_m^2 + (\Delta\lambda)^2} \tag{S7}$$

If now all diameters in equation (S7) are shifted for $e^{-\frac{s^2}{2}}$, we obtain:

$$D_m^3 = K_2 \frac{\left(D_m e^{-\frac{s^2}{2}}\right)^5}{\left(D_m e^{-\frac{s^2}{2}}\right)^2 + (\Delta\lambda)^2} \tag{S8}$$

Using (S6) in (S8) in order to eliminate factors $e^{-\frac{s^2}{2}}$ we obtain:

$$D_m^3 = \frac{K_2^{-\frac{2}{3}} D_m^5 \left(\frac{\lambda_{SPR}}{\lambda_0}\right)^{15}}{K_2^{-\frac{2}{3}} D_m^2 \left(\frac{\lambda_{SPR}}{\lambda_0}\right)^6 + (\Delta\lambda)^2} \tag{S9}$$

Solving (S9) by variable $D_m$, analytical expression is obtained:

$$D_m = K^{\frac{1}{3}} \left(\frac{\lambda_0}{\lambda_{SPR}}\right)^3 \frac{\Delta\lambda}{\sqrt{\left(\frac{\lambda_{SPR}}{\lambda_0}\right)^9 - 1}} \tag{S10}$$

Final formula is shifted from (S10) by $\left(\frac{\lambda_{SPR}}{\lambda_0}\right)^2$ so finally it writes:

$$D_m^* = K^{\frac{1}{3}} \left(\frac{\lambda_0}{\lambda_{SPR}}\right) \frac{\Delta\lambda}{\sqrt{\left(\frac{\lambda_{SPR}}{\lambda_0}\right)^9 - 1}} \tag{S11}$$
